# Health literacy of patients admitted for elective surgery

**DOI:** 10.1007/s10389-016-0774-z

**Published:** 2016-12-06

**Authors:** E. S. Koster, A. Schmidt, D. Philbert, E. M. W. van de Garde, M. L. Bouvy

**Affiliations:** 1 Division of Pharmacoepidemiology and Clinical Pharmacology, Utrecht University, PO Box 80082 3508 TB, Utrecht, The Netherlands; 2 Department of Clinical Pharmacy, St. Antonius Hospital Nieuwegein, Utrecht, The Netherlands

**Keywords:** Health literacy, Communicative, Critical, Functional, FCCHL, Hospital, Surgery

## Abstract

**Aim:**

Patients with limited health literacy have poorer surgical outcomes. However, current studies assessing the prevalence of limited health literacy in patients expecting surgery are small scale. We aimed to provide insight into the health literacy level of patients undergoing planned surgery.

**Subject and Methods:**

Patients aged ≥18 years visiting the preoperative screening department were approached in the waiting area and invited to participate in a brief interview including the Functional Communicative Critical Health Literacy (FCCHL).

**Results:**

In total, 225 patients (84.9% response) were studied. Based on the FCCHL, 37.3% of the patients were classified as having limited health literacy. The mean score in the critical domain (2.7 ± 0.9) was lower than scores in the functional (3.3 ± 0.6) and communicative (3.3 ± 0.6) domains.

**Conclusion:**

More than one third of the patients admitted to the hospital for surgery had limited health literacy. Healthcare professionals should be aware of the different health literacy levels and tailor their information provision strategies accordingly.

## Introduction

Health literacy, defined as the ability to obtain, understand and use information in health-related decisions, can be described in terms of functional, communicative and critical skills (Nutbeam 2008, 2009). Functional skills are necessary for reading and writing in order to function in everyday situations, communicative skills are more advanced skills to extract and apply (new) information in different situations, and critical skills are needed for analyzing and reflecting on information or advice.

Several studies have shown that patients with limited health literacy have poorer health outcomes (Berkman et al. 2011). Patients who undergo surgery often receive specific preoperative instructions, e.g., with respect to food and drink restrictions or temporary discontinuation of medication. Chew et al. (2004) showed that patients with limited health literacy were less likely to comply with preoperative instructions, which could lead to delays, cancellation of surgical procedures or even negative surgical outcomes.

To date, only a few small studies have been performed on the prevalence and consequences of limited health literacy in perioperative care for surgical patients (Beitler et al. 2010; Choi 2013; De Oliveira et al. 2015; Gordon and Wolf 2009). Therefore, we aimed to provide insight into the health literacy level of patients admitted to the hospital for elective surgery.

## Methods

### Design and setting

We conducted a cross-sectional study at the preoperative screening department of the St. Antonius Hospital (850-bed teaching hospital) in Nieuwegein, The Netherlands. Before planned hospital admission, patients visit this department and receive information about the surgical procedure, anesthesia and the general procedure for hospital admission including preoperative instructions (e.g., food or drink restrictions or medication instructions).

The study protocol was approved by the Institutional Review Board of the Division of Pharmacoepidemiology & Clinical Pharmacology, Utrecht University, and the local review committee of St. Antonius Hospital, Nieuwegein.

### Study population

Patients aged ≥18 years who were scheduled for surgery and visiting the preoperative screening department were invited to participate in the study. Patients insufficiently proficient in the Dutch language and patients with dementia were excluded from the study. Patients who signed the informed consent form were interviewed to assess their health literacy level. A master pharmacy student of the Utrecht School of Pharmacy performed the interviews.

### Data collection

Data were collected from September–November 2015. The Dutch version of the Functional Communicative Critical Health Literacy (FCCHL) instrument, a validated questionnaire to assess health literacy skills (Nutbeam 2008, 2009; van der Vaart et al. 2012), was used to guide data collection (Ishikawa et al. 2008). The FCCHL measures three aspects of health literacy using 14 questions, namely functional (5 questions), communicative (5 questions) and critical skills (4 questions) (van der Vaart et al. 2012). All questions were scored on a four point Likert-scale (1–4) ranging from never perceiving difficulties to often perceiving difficulties. Mean total FCCHL scores and mean sub-scale scores were calculated by summing item scores divided by the total number of items (in total or in the sub-scale), resulting in a score ranging from 1 (low health literacy) to 4 (high health literacy). Based on previous research, patients with scores ≤3 in total or on a sub-scale were defined as having limited health literacy (Fransen et al. 2011). In addition, information about socio-demographic characteristics such as age, gender, educational level and ethnic origin was collected during the interview.

### Data analysis

Descriptive statistics were used to calculate health literacy in total and for the three different sub-scales (functional, communicative and critical). We presented the proportion of patients with an FCCHL mean total and sub-scale score ≤3 (see above). Logistic regression analysis controlling for age, gender, educational level and ethnic background was used to calculate odds ratios and their corresponding 95% confidence intervals (CIs) for the association between health literacy and patient characteristics. The possibility for a predictive model for limited health literacy was also investigated. All analyses show the odds ratios for limited health literacy compared with adequate health literacy. Data were analyzed using IBM SPSS version 23.0 (IBM Corp., Armonk, NY, USA) for Windows.

## Results

### Study population

In total, 225 patients (84.9%) out of 262 invited patients agreed to participate. The most common reasons for non-willingness to participate were lack of interest in the study (18.9%, n = 7) and lack of time (21.6%, n = 8). Table 1 shows the study population characteristics. The majority of the participants were of native Dutch origin (84.0%).

**Table 1:** Study population characteristics

	Study population (N = 225)
Female gender, % (n)	55.1 (124)
Mean age, years (SD)	56.4 (15.1)
Ethnicity*, % (n)	
Native Dutch	87.6 (197)
Western immigrant	8.4 (19)
Non-Western immigrant	4.0 (9)
Educational level**, % (n)	
Low	33.8 (76)
Middle	36.4 (82)
High	29.8 (67)

*Ethnic origin was classified into three groups: native (Dutch), non-Western immigrant [someone whose country of origin is or lies in Turkey, Africa, Latin America or Asia, with the exception of Indonesia (or the former Dutch East Indies) and Japan] or Western immigrant.

**Low educational level was defined as no secondary education (only primary school) or a lower vocational level. Middle was defined as a higher secondary education or intermediate vocational level, and high educational level was defined as a higher vocational or university level.

### Health literacy skills

**Fig. 1:**
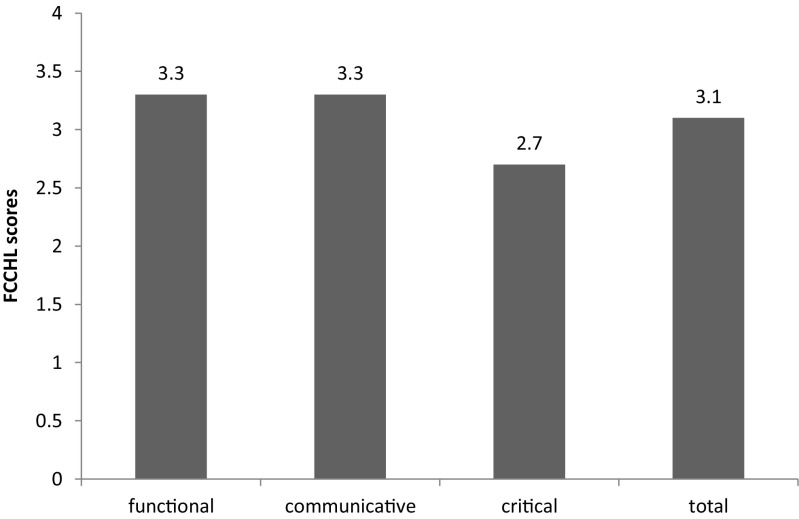
Mean FCCHL scores

Overall mean scores ≤3 were found in 84 patients (37.3%), indicating limited health literacy. For the three different sub-scales, 30.7% (n = 69), 31.6% (n = 71) and 54.7% (n = 123) scored below the threshold of three in the functional, communicative and critical domains, respectively. Mean FCCHL scores were lowest for critical health literacy (mean score: 2.7 ± 0.9) compared to functional and communicative health literacy (mean score: 3.3 ± 0.6 for both domains) (Fig. 1).

As shown in Table 2, advancing age, non-Western background and lower educational level were significantly associated with limited health literacy (p < 0.05). A separate multivariate logistic regression was performed for each of the three domains (functional, communicative and critical health literacy; Appendix 1 (Tables 3, 4 and 5)). Although not all domains showed the same significant associations, they showed similar trends for characteristics associated with limited health literacy.

**Table 2:** Patient characteristics associated with limited health literacy

	Adequate health literacy*	Limited health literacy*	Crude OR (95% CI)	Adjusted** OR (95% CI)	*p*
	N = 141	N = 84			
Age, mean (SD)	54.6 (14.9)	59.4 (15.1)	1.02 (1.0–1.0)	1.02 (1.0–1.0)	0.03
Gender, % (n)					
Male	40.4 (57)	52.4 (44)	1.6 (0.9–2.8)	1.6 (0.9–2.9)	0.12
Female	59.6 (84)	47.6 (40)	REF	REF	REF
Educational level, % (n)					
Low	29.1 (41)	41.7 (35)	2.7 (1.3–5.6)	2.5 (1.1–5.3)	0.02
Middle	34.8 (49)	39.3 (33)	2.1 (1.1–4.4)	2.5 (1.2–5.4)	0.02
High	36.2 (51)	19.0 (16)	REF	REF	REF
Non-Western background, % (n)	4.3 (6)	15.5 (13)	4.1 (1.5–11.3)	6.0 (2.1–17.8)	0.001

*Adequate health literacy was defined as a total FCCHL score >3 and limited health literacy as a total FCCHL score ≤3

**Adjusted for age, gender, educational level and background

REF = reference category

Characteristics that were significantly associated in the overall multivariate logistic regression were added to a predictive model for limited health literacy (regression equation: −2.9 + [0.003*age] + [1.8*non-Western background] + [0.83*low education level] + [0.90*middle education level]), which showed a good fit (Hosmer and Lemeshow test p = 0.74); however, the predictive power of the model was poor (area under the curve: 0.68).

## Discussion

More than one third of patients scheduled for elective surgery were classified as having limited health literacy. The prevalence of limited health literacy found in our study is in line with other studies (van der Heide et al. 2015). Also, as in previous studies, patients scored lowest in the critical domain (Heijmans et al. 2015; Ishikawa et al. 2009). Patients may be able to read and write, but may still be hampered in more complex tasks or decisions as reflected by the lower scores in this domain. Heijmans et al. (2015) reported that mainly communicative and critical health literacy is important for successful disease self-management. Thus, a broad range of skills is necessary for active patient participation and optimal outcomes.

In addition, we demonstrated associations between limited health literacy and demographic factors, e.g., advancing age, lower educational level and non-native (non-Western) background. This is in line with findings reported in the literature (Chew et al. 2004; Koster et al. 2015; van der Heide et al. 2015). However, the predictive power of our model was not very high, suggesting that healthcare professionals cannot solely depend on age, education level and non-Western background to identify patients at risk of having limited health literacy.

One of the strengths of the study is that the health literacy screening was conducted verbally, thereby also enabling patients who experience difficulties in reading or writing, and thus might have limited functional health literacy, to participate. In addition, selection bias is unlikely due to the high response rate (>80%). Many previous studies have used health literacy measurement instruments that focus solely on functional skills, e.g., reading comprehension, while the FCCHL we used in this study also includes assessment of communicative and critical skills and thus gives insight into the broader range of health literacy skills. However, as the FCCHL is a subjective instrument based on self-reporting, it may lead to observations that differ from actual patient behavior. In real-life situations people may experience even more difficulties with certain tasks, in which case our results underestimate the problem of limited health literacy.

Low health literacy has previously been associated with poorer surgical outcomes and misunderstanding of information (Koster et al. 2015). Inadequate understanding of information or instructions may also have negative consequences for the hospital in terms of delayed or cancelled surgical procedures. This study shows that a considerable proportion of patients who are undergoing surgery have limited health literacy.

The first step in tackling this problem is creating awareness among healthcare professionals. They should take into account the different skill levels of their patients and tailor their information and communication strategies to suit patients’ individual needs. Many current health literacy interventions consist of handing out (additional) written information or providing information in another language (Taggart et al. 2012; Wali et al. 2015). However, patients with limited health literacy may have more complex problems, as shown by the lower scores in the critical domain in our study, hampering active patient participation during consultations and treatment decisions. Therefore, healthcare providers should ensure they provide easy–to-understand information and instructions. Checking comprehension using teach-back methods, for example, can reduce misunderstandings and potentially prevent negative health outcomes (Samuels-Kalow et al. 2016).

In conclusion, this study shows that a considerable proportion of patients scheduled for surgery have limited health literacy. Patients scored lowest in the critical domain, which may have consequences for self-management. Healthcare providers involved in preoperative screening should actively identify patients with poor health literacy and should adapt their information and instructions for these patients.

## Appendix 1

**Table 3:** Multivariate logistic regression for FCCHL subscale Functional Health Literacy

	Adequate health literacy*	Limited health literacy*	Crude OR (95% CI)	Adjusted** OR (95% CI)	*p*
	N = 141	N = 84			
Age, mean (SD)	55.2 (15.2)	59.0 (14.8)	1.02 (1.0–1.0)	1.03 (1.0–1.1)	0.03
Gender, % (n)					
Male	42.3 (66)	50.7 (35)	1.4 (0.8–2.5)	1.3 (0.7–2.5)	0.36
Female	57.7 (90)	49.3 (34)	REF	REF	REF
Educational level, % (n)					
Low	32.7 (51)	36.2 (25)	1.6 (0.7–3.3)	1.3 (0.6–2.9)	0.50
Middle	34.6 (54)	40.6 (28)	1.7 (0.8–3.4)	1.9 (0.9–4.1)	0.10
High	32.7 (51)	23.2 (16)	REF	REF	REF
Non-Western background, % (n)	3.8 (6)	18.8 (13)	5.8 (2.1–16.0)	8.2 (2.8–23.8)	0.000

*Adequate health literacy was defined as a total FCCHL score >3 and limited health literacy as a total FCCHL score ≤3

**Adjusted for age, gender, educational level and background

**Table 4:** Multivariate logistic regression for FCCHL subscale Communicative Health Literacy

	Adequate health literacy* N = 141	Limited health literacy* N = 84	Crude OR (95% CI)	Adjusted** OR (95% CI)	*p*
Age, mean (SD)	54.4 (14.6)	60.6 (15.4)	1.03 (1.0–1.1)	1.03 (1.0–1.0)	0.04
Gender, % (n)					
Male	40.3 (62)	54.9 (39)	1.8 (1.0–3.2)	1.7 (0.9–3.2)	0.08
Female	59.7 (92)	45.1 (32)	REF	REF	REF
Educational level, % (n)					
Low	27.9 (43)	46.5 (33)	3.5 (1.6–7.6)	3.1 (1.4–7.0)	0.007
Middle	36.4 (56)	36.6 (26)	2.1 (1.0–4.6)	2.5 (1.1–5.5)	0.03
High	35.7 (55)	16.9 (12)	REF	REF	REF
Non-Western background, % (n)	6.5 (10)	12.7 (9)	2.1 (0.8–5.4)	3.0 (1.1–8.4)	0.04

*Adequate health literacy was defined as a total FCCHL score >3 and limited health literacy as a total FCCHL score ≤3

**Adjusted for age, gender, educational level and background

**Table 5:** Multivariate logistic regression for FCCHL subscale Critical Health Literacy

	Adequate health literacy*	Limited health literacy*	Crude OR (95% CI)	Adjusted** OR (95% CI)	*p*
	N = 141	N = 84			
Age, mean (SD)	52.8 (14.4)	59.4 (15.1)	1.03 (1.0–1.1)	1.03 (1.0–1.1)	0.05
Gender, % (n)					
Male	39.2 (40)	49.6 (61)	1.5 (0.9–2.6)	1.4 (0.8–2.4)	0.30
Female	60.8 (62)	50.4 (62)	REF	REF	REF
Educational level, % (n)					
Low	29.1 (41)	41.7 (35)	2.1 (1.1–4.1)	1.7 (0.8–3.4)	0.16
Middle	34.8 (49)	39.3 (33)	1.3 (0.7–2.4)	1.4 (0.7–2.8)	0.30
High	36.2 (51)	19.0 (16)	REF	REF	REF
Non-Western background, % (n)	4.9 (5)	11.4 (14)	2.5 (0.9–7.2)	3.5 (1.2–10.5)	0.03

*Adequate health literacy was defined as a total FCCHL score >3 and limited health literacy as a total FCCHL score ≤3

**Adjusted for age, gender, educational level and background
